# Nutrition and Respiratory Health—Feature Review

**DOI:** 10.3390/nu7031618

**Published:** 2015-03-05

**Authors:** Bronwyn S. Berthon, Lisa G. Wood

**Affiliations:** Centre for Asthma and Respiratory Diseases, Level 2, Hunter Medical Research Institute, University of Newcastle, Lot 1 Kookaburra Circuit, New Lambton Heights, NSW 2305, Australia; E-Mail: bronwyn.berthon@newcastle.edu.au

**Keywords:** respiratory disease, asthma, COPD, dietary patterns, antioxidants, vitamin C, vitamin E, flavonoids, vitamin D, obesity, adipokines, undernutrition

## Abstract

Diet and nutrition may be important modifiable risk factors for the development, progression and management of obstructive lung diseases such as asthma and chronic obstructive pulmonary disease (COPD). This review examines the relationship between dietary patterns, nutrient intake and weight status in obstructive lung diseases, at different life stages, from *in-utero* influences through childhood and into adulthood. *In vitro* and animal studies suggest important roles for various nutrients, some of which are supported by epidemiological studies. However, few well-designed human intervention trials are available to definitively assess the efficacy of different approaches to nutritional management of respiratory diseases. Evidence for the impact of higher intakes of fruit and vegetables is amongst the strongest, yet other dietary nutrients and dietary patterns require evidence from human clinical studies before conclusions can be made about their effectiveness.

## 1. Introduction

Diet and nutrition are increasingly becoming recognised as modifiable contributors to chronic disease development and progression. Considerable evidence has emerged indicating the importance of dietary intake in obstructive lung diseases such as asthma and chronic obstructive pulmonary disease (COPD) in both early life and disease development [1,2] and management of disease progression [3,4]. These respiratory diseases are characterised by airway and systemic inflammation, airflow obstruction, deficits in lung function and significant morbidity and mortality, as well as being costly economic burdens [5,6]. Pharmacological management remains the mainstay for treatment of respiratory diseases, and while treatment options are advancing, dietary intake modification could be an important adjuvant to disease management and an important consideration for disease prevention. Dietary patterns as well as intake of individual nutrients have been evaluated in observational and experimental studies throughout life stages and disease stages to elucidate their role in respiratory diseases. This review concentrates on evidence regarding the role of dietary patterns, individual nutrients, weight status and adipokines in asthma and COPD.

## 2. Dietary Intake and Respiratory Diseases

### 2.1. Dietary Patterns

Various dietary patterns have been linked to the risk of respiratory disease [7]. The Mediterranean diet has been found to have protective effects for allergic respiratory diseases in epidemiological studies [8]. This dietary pattern consists of a high intake of minimally processed plant foods, namely; fruit, vegetables, breads, cereals, beans, nuts and seeds, low to moderate intake of dairy foods, fish, poultry and wine and low intake of red meat. High intakes of olive oil result in a dietary composition that is low in saturated fat though still moderate in total fat. In children, several studies showed that adherence to the Mediterranean diet is inversely associated with atopy and has a protective effect on atopy, wheezing and asthma symptoms [9,10,11]. The Mediterranean diet may also be important for maternal diet, as a study in Spain found that a high Mediterranean diet score during pregnancy was protective for persistent wheeze and atopic wheeze in children at 6.5 years of age [12]. Though one cross-sectional study in Japan reported a strong association between the adherence to the Mediterranean diet and asthma control [13], there is less evidence available to support this dietary pattern in adults.

The “western” dietary pattern, prevalent in developed countries, is characterised by high consumption of refined grains, cured and red meats, desserts and sweets, french fries, and high-fat dairy products [2,14]. This pattern of intake has been associated with increased risk of asthma in children [15,16]. Furthermore, in children, increased intake of fast food such as hamburgers and related eating behaviours, for example salty snack eating and frequent take away consumption, are correlated with the presence of asthma, wheezing and airway hyperresponsiveness (AHR) [17,18]. In adults, a western diet has been shown to be positively associated with increased frequency of asthma exacerbation [19], but not related to asthma risk. In addition, an acute challenge with a high fat fast food meal has been shown to worsen airway inflammation [20]. While this dietary pattern appears to be deleterious in children and adults with asthma, studies examining the effect of this dietary pattern in maternal diets have found no relationship with a consumption of a “western” style diet in pregnancy and risk of asthma in offspring [21]. Cross-sectional studies have also found that the “western” diet is associated with an increased risk of COPD [2]. In summary the Mediterranean diet appears to be protective in children, though there is less evidence for benefits in the maternal diet and in adults. There is evidence to suggest that a “western” style dietary pattern increases risk of asthma in children, has worse outcomes for adults with asthma and is related to COPD risk.

### 2.2. Fruit and Vegetables

Fruit and vegetable intake has been investigated for potential benefits in association with respiratory conditions due to their nutrient profile consisting of antioxidants, vitamins, minerals, fibre and phytochemicals. The mechanisms by which the nutrients in fruit and vegetables exert beneficial effects in respiratory conditions are discussed in the sections below. Epidemiological evidence reviewed by Saadeh *et al.* [7] showed that fruit intake was associated with a low prevalence of wheezing and that cooked green vegetable intake was associated with a low prevalence of wheezing and asthma in school children aged 8–12 years old. Furthermore low vegetable intake in children was related to current asthma [7]. In adults, Grieger *et al.* [22] discusses the heterogeneous nature of the data describing fruit and vegetable intake and lung function, with one study showing no effect on lung function of higher fruit and vegetable intake over 10 years [23], yet in another study, increased fruit intake over 2 years was associated with increased FEV_1_ [23], while another study showed that a large decrease in fruit intake over 7 years was associated with decreased FEV_1_ [24]. We recently conducted an intervention in adults with asthma and found that subjects who consumed a high fruit and vegetable diet for 3 months, had a decreased risk of asthma exacerbation, compared to subjects who consumed a low fruit and vegetable diet [25]. A recent meta-analysis of adults and children, which analysed 12 cohorts, 4 population-based case-control studies, and 26 cross-sectional studies provides important new evidence showing that a high intake of fruit and vegetables reduces the risk of childhood wheezing, and that fruit and vegetable intake is negatively associated with asthma risk in adults and children [26]. While some studies of maternal diet have found no relationship with fruit and vegetable intake and asthma in children [27], other studies have found that increased fruit and vegetable intake were related to a decreased risk of asthma in children [21,28]. Increased fruit and vegetable intake may be protective against COPD development, with consumption of a “prudent” diet including increased fruit and vegetables being protective against lung function decline [3]. Two randomized controlled trials (RCT’s) manipulating fruit and vegetable intake have been conducted in COPD. A 12 week study showed no effect of a high fruit and vegetable intake on FEV1, systemic inflammation or airway oxidative stress [29]. However, a 3-year study in 120 COPD patients revealed an improvement in lung function in the high fruit and vegetable group compared to the control group [30], suggesting that longer term intervention is needed to provide a therapeutic effect. There is considerable evidence to suggest that a high intake of fruit and vegetables is favourable for all life stages of asthma and evidence is emerging which suggests the same in COPD.

### 2.3. Omega-3 Fatty Acids and Fish

Omega-3 polyunsaturated fatty acids (PUFA) from marine sources and supplements have been shown to be anti-inflammatory through several cellular mechanisms including their incorporation into cellular membranes and resulting altered synthesis of eicosanoids [31]. Experimental studies have shown that long chain omega-3 PUFA’s decrease inflammatory cell production of pro-inflammatory prostaglandin (PG) E_2_, leukotriene (LT) B_4_ [32] and activity of nuclear factor-kappaB (NF-κB), a potent inflammatory transcription factor [33]. Long chain omega-3 PUFA’s also down regulate pro-inflammatory cell cytokine production (interleukin-1β (IL-1β), tumor necrosis factor-α (TNF-α)) by monocytes and macrophages, decrease expression of cellular adhesion molecules on monocytes and endothelial cells and reduce production of ROS in neutrophils [34]. Saddeh *et al.* [7] reported that the evidence describing the relationship between omega-3 PUFA’s or fish consumption and respiratory conditions in childhood is contradictory. Some observational studies show that intake of oily fish is negatively associated with AHR and asthma [35,36]. However, evidence from Japan suggests that frequency of fish consumption is positively related to asthma risk [37] and in Saudi Arabia fish intake was not related to the presence of asthma or wheezing at all [18]. Similarly in adults, the data is heterogeneous, with omega-3 PUFAs or fish being associated with improved lung function [38] and decreased risk of asthma [39], AHR [35] and wheeze [36] in some, but not all studies [40]. Maternal dietary intake of oily fish was found to be protective of asthma in children 5 years of age if born to mothers with asthma [41] and a recent systematic review of omega-3 fatty acid supplementation studies in women during pregnancy found that the risk of asthma development in children was reduced [42]. The data examining the possible benefits of dietary omega-3 fatty acid supplementation in asthma are heterogeneous and as summarized by a 2002 Cochrane review [43], to date there is insufficient evidence to recommend omega-3 PUFA supplementation in asthma. Omega-3 PUFA may have positive effects in COPD, as higher levels of DHA in serum were found to decrease the risk of developing COPD [44]. Experimental studies in humans with COPD including supplementation with omega-3 found lower levels of TNF-α [45] and improved rehabilitation outcomes [46], though no improvements were seen in FEV_1_. Several studies using omega-3 PUFA supplementation in COPD are currently underway and will provide important new information to inform the field [47,48,49]. Consumption of oily fish or supplementation with omega-3 PUFA’s may have positive effects in asthma and COPD, though strong evidence to support the experimental and epidemiological data is not yet available.

## 3. Nutrients and Respiratory Disease

### 3.1. Antioxidants and Oxidative Stress

Dietary antioxidants are an important dietary factor in protecting against the damaging effects of oxidative stress in the airways, a characteristic of respiratory diseases [50]. Oxidative stress caused by reactive oxygen species (ROS), is generated in the lungs due to various exposures, such as air pollution, airborne irritants and typical airway inflammatory cell responses [51]. Also, increased levels of ROS generate further inflammation in the airways via activation of NF-κB and gene expression of pro-inflammatory mediators [52]. Antioxidants including vitamin C, vitamin E, flavonoids and carotenoids are abundantly present in fruits and vegetables, as well as nuts, vegetable oils, cocoa, red wine and green tea. Dietary antioxidants may have beneficial effects on respiratory health, from influences of the maternal diet on the fetus, and intake in children through to adults and pregnant women with asthma and adults with COPD.

α-tocopherol is a form of vitamin E, which helps maintain integrity of membrane fatty acids, by inhibiting lipid peroxidation [22]. Carotenoids are plant pigments and include; α- and β-carotene, lycopene, lutein and β-cryptoxanthin. This group of fat soluble antioxidants have been shown to benefit respiratory health due to their ability to scavenge ROS and reduce oxidative stress [22]. The antioxidant lycopene, present predominantly in tomatoes, may be beneficial in respiratory conditions, indeed lycopene intake has been positively correlated with FEV_1_ in both asthma and COPD [53] and an intervention study in asthma showed that lycopene supplementation could suppress neutrophilic airway inflammation [54].

Antioxidants may also be important in asthma during pregnancy, as while oxidative stress commonly increases during normal pregnancies, in women with asthma oxidative stress is heightened [55]. During pregnancy there is a compensatory increase in circulating and placental antioxidants in asthma *versus* women without asthma, to protect the foetus against damaging effects of oxidative stress [55,56]. Improving antioxidant intake in pregnant women with asthma may be beneficial as poor fetal growth outcomes are associated with low levels of circulating antioxidants and dietary antioxidants are the first defense mechanism against ROS [22]. Maternal intake of vitamin E, vitamin D, milk, cheese and calcium during pregnancy are negatively associated, while vitamin C is positively associated, with wheezing in early childhood [57,58]. Antioxidants including lycopene appear to have positive influences in respiratory conditions, further detail is provided below on evidence for vitamin C, vitamin E and flavonoids and their role in the maternal diet, diets of children and adults with asthma and adults with COPD.

### 3.2. Vitamin C

Vitamin C has been enthusiastically investigated for benefits in asthma and links to asthma prevention. *In vitro* data from endothelial cell lines showed that vitamin C could inhibit NF-κB activation by IL-1, TNF-α and block production of IL-8 via mechanisms not dependent on the antioxidant activity of vitamin C [59]. Anti-inflammatory and anti-asthmatic effects of vitamin C supplementation *in vivo*, have been shown through allergic mouse models of asthma. Jeong *et al.* [60] reported decreased AHR to methacholine and inflammatory cell infiltration of perivascular and peribronchiolar spaces when vitamin C was supplemented during allergen challenge. While Chang *et al.* [61] found that high dose Vitamin C supplementation in allergen challenged mice decreased eosinophils in BALF and increased the ratio of Th1/Th2 cytokine production shifting the inflammatory pattern to Th1 dominant. Observational studies in children showed consumption of fruit, a rich source of vitamin C, was related to reduced wheezing [62] and vitamin C intake was negatively associated with wheezing [63], while another study reported no relationship between vitamin C intake and lung function [64]. Grieger *et al.* [22] also reported conflicting evidence for effects of vitamin C intake in adults, with epidemiological studies showing a positive association between vitamin C intake and lung function in some [65], but not all studies [23,66]. Despite the observational data linking vitamin C to lung health, supplementation with vitamin C has not been shown to reduce the risk of asthma [66] which may be related to the interdependence of nutrients found in foods, resulting in lack of efficacy when supplementing with isolated nutrients. Evidence from experimental and observational studies suggests that Vitamin C might be important in COPD pathogenesis and management. Koike *et al.* [67] reported that in knock out mice unable to synthesize vitamin C, vitamin C supplementation was able to prevent smoke induced emphysema and also to restore damaged lung tissue and decrease oxidative stress caused by smoke induced emphysema. A case control study in Taiwan reported that subjects with COPD had lower dietary intake and lower serum levels of vitamin C than healthy controls [68]. Indeed an epidemiological study in the United Kingdom of over 7000 adults aged 45–74 years found that increased plasma vitamin C concentration was associated with a decreased risk of obstructive airways disease, suggestive of a protective effect [69]. Thus, in summary, while observational data has suggested that vitamin C is important for lung health, intervention trials showing efficacy are lacking and it appears that supplementation with vitamin C-rich whole foods, such as fruit and vegetables may be more effective.

### 3.3. Vitamin E

The vitamin E family comprises of 4 tocopherols and 4 tocotrienols, with the most plentiful in the diet or in tissues being α-tocopherol and γ-tocopherol [70]. Vitamin E works synergistically with vitamin C, as following neutralisation of ROS, oxidised vitamin E isoforms can be processed back into their reduced form by vitamin C [71]. Abdala-Valencia *et al.* [72] discuss the evidence for the roles of α-tocopherol and γ-tocopherol in allergic lung inflammation in mechanistic animal studies and clinical trials. Supplementation of mice with α-tocopherol reduced allergic airway inflammation and AHR [73], while γ-tocopherol was pro-inflammatory and augmented AHR, negating the positive effects of α-tocopherol [74]. Other animal studies report that γ-tocopherol may assist in resolving inflammation caused by ozone exposure and endotoxin induced neutrophilic airway inflammation, owing to its ability to oxidize reactive nitrogen species [75,76]. A study in humans showed that both α and γ-tocopherol may be effective in decreasing LPS induced neutrophilic inflammation [77]. The conflicting results from these supplementation studies are likely to be influenced by baseline tissue levels of vitamin E [72], with α-tocopherol supplementation leading to improved lung function and wheeze in Europe, where γ-tocopherol levels are low [78,79,80], but not in the US, where γ-tocopherol intake is high due to soybean oil consumption [81,82,83]. As a result, meta-analysis of vitamin E effects on asthma outcomes is equivocal; it is likely that supplementation with physiological concentrations of α-tocopherol in the context of a background diet low in γ-tocopherol, may be most beneficial in asthma and further research testing this hypothesis is required. In COPD, serum levels of vitamin E have been shown to be decreased during exacerbation, which suggests increased intake may be helpful to improve vitamin E concentrations [84]. Vitamin E has been shown to reduce biomarkers of oxidative stress in adults with COPD in one RCT [85], but not another [86]. In the Women’s Health Study (*n* = 38,597), the risk of developing chronic lung disease over a 10 year supplementation period was reduced by 10% in women using vitamin E supplements (600 IU on alternate days) [87].

Dietary intake of vitamin E lower than recommended dietary intakes has been reported in pregnant women with a family history of allergic disease [88] and recent work in animal models has highlighted α-tocopherol may be important for allergic mothers in pregnancy. Allergic female mice were supplemented with α-tocopherol prior to mating and following allergen challenge the offspring showed reduced response to allergen challenge with decreased eosinophils in BALF [89]. The offspring also showed reduced development of lung dendritic cells, necessary for producing allergic responses. Evidence from observational studies also suggests that reduced maternal dietary intake of vitamin E is related to an increased risk of childhood asthma and wheeze [90,91,92] and increased *in vitro* proliferative responses in cord blood mononuclear cells (CBMC) [93]. A mechanistic study by Wassall *et al.* [94] examined the effect of α-tocopherol and vitamin C on CBMC and maternal peripheral blood mononuclear cells (PBMC). α-tocopherol was mostly anti-inflammatory, although increased proliferation and increased TGF-β were seen with some allergens. However, the addition of vitamin C to the system had inflammatory actions, with increased production of pro-inflammatory cytokines, combined with reduced production of IL-10 and TGF-β. This study by Wassall *et al.* [94] demonstrates that supplementation with these antioxidants does modulate immune responses in pregnancy, however several of the results are unexpected, highlighting the complex nature of the relationships between dietary nutrients and disease. In asthma the experimental data for vitamin E are compelling, yet supplementation benefits are not well described. In COPD there is currently not enough evidence to make conclusions about vitamin E supplementation.

### 3.4. Flavonoids

Flavonoids are potent antioxidants and have anti-inflammatory as well as anti-allergic actions due in part, to their ability to neutralise ROS [95]. There are 6 classes of flavonoids including flavones, flavonols, flavanones, isoflavones and flavanols [96], which are widely distributed throughout the diet and found in fruit, vegetables, nuts, seeds, stems, flowers, roots, bark, dark chocolate, tea, wine and coffee [96]. Tanaka *et al.* [95] present the evidence for the benefits of dietary flavonoids in asthma development and progression. In addition to reducing oxidative stress, *in vitro* experiments have found that many individual flavonoids have inhibitory effects on IgE mediated immune responses such as histamine secretion by mast cells, shift in cytokine production from Th-2 to Th-1 production and decreased NF-κB activation and inhibition of TNF-α [97,98,99,100]. Experimental studies of flavonoids in animal models of allergic asthma have shown reduced airway and peripheral blood inflammation, decreased bronchoconstriction and AHR and lower eosinophils in BALF, blood and lung tissue [101,102,103,104]. In humans, evidence from a case control study in adults showed that apple and red wine consumption, rich sources of flavonoids, was associated with reduced asthma prevalence and severity [66]. However a follow-up study investigating intake of 3 subclasses of flavonoids did not find any associations with asthma prevalence or severity [105]. There are a limited number of experimental studies using flavonoid supplements in humans with asthma. Three RCT’s in adults with asthma using a product called pycnogenol, which contains a mixture of bioflavonoids, reported benefits including increased lung function, decreased symptoms and reduced need for rescue inhalers [106]. There is a paucity of evidence for the effects of flavonoids in the maternal diet and respiratory outcomes in children. One study which found a positive association of maternal apple intake and asthma in children at 5 years, suggests that the flavonoid content of apples may be responsible for the beneficial relationship [107]. Evidence for the effects of flavonoids in respiratory conditions is emerging and promising. Though like vitamin C, it may be difficult to disentangle the effects of flavonoids from other nutrients in flavonoid-rich foods. Supplementation of individual flavonoids in experimental animal studies has provided evidence to suggest that intervention trials in humans may be warranted.

### 3.5. Vitamin D

Epidemiological studies show promising associations between vitamin D and lung health; however the mechanisms responsible for these effects are poorly understood. Vitamin D can be obtained from dietary sources or supplementation; however sun exposure is the main contributor to vitamin D levels [108]. While vitamin D has beneficial effects independent of UV exposure [109], it can be difficult to separate this potential confounder from direct effects of vitamin D on lung health [110]. The review by Foong and Zosky [111] presents the current evidence for the role of vitamin D deficiency in disease onset, progression and exacerbation in respiratory infections, asthma and COPD. Respiratory infections contribute to disease progression and exacerbation in both COPD and asthma. Vitamin D appears to have a protective role against the susceptibility to and severity of these infections [111], as active vitamin D (1,25 (OH)_2_D) modifies production of antimicrobial cathelicidins and defensins that kill bacteria and induce wound repair [112]. Activated vitamin D also decreases the expression of rhinovirus receptors in endothelial cell cultures and PBMC’s [113]. *In vitro* studies also support the link between vitamin D and airway remodelling as active vitamin D inhibits airway smooth muscle (ASM) cell proliferation [114] and deficiency impairs normal lung development [115]. Furthermore, animal models suggest that vitamin D can inhibit Th1 and Th2 cell cytokine production [116]. Epidemiological evidence links low levels of vitamin D with wheeze and respiratory infections, though evidence for the link with asthma onset is weak and inconsistent [111]. In children, low circulating vitamin D was related to lower lung function, increased corticosteroid use and exacerbation frequency [117]. Also in children with steroid resistant asthma, low vitamin D was related to increased ASM thickness [117]. Other observational studies report that in children, low levels of vitamin D are associated with asthma exacerbation [118]. Several observational studies support the role of vitamin D for protection against respiratory conditions in children. Zosky *et al.* [119] found that vitamin D deficiency at 18 weeks gestation was associated with lower lung function and current wheeze in children 6 years of age and an increased risk of asthma in boys. The role for vitamin D in enhancing steroid responsiveness suggested by observational studies [120] is supported by mechanistic studies [121], and in concert with the actions of vitamin D in infection, may explain the effect of vitamin D in reducing asthma exacerbations [111]. Only one intervention trial has been conducted using vitamin D in adults with asthma, which found that rate of first exacerbation was reduced in subjects who demonstrated an increase in circulating vitamin D3 following supplementation [122]. Data for the role of vitamin D in COPD onset is limited, though several cross-sectional studies have reported an association between low vitamin D levels, or deficiency, with COPD incidence [123]. Blood vitamin D levels have also been correlated with lung function in COPD patients [124,125]. Experimental data suggest that vitamin D may be important in COPD for its effect on normal lung growth and development, though human data to support this is not available. It is possible that COPD onset may also be impacted by cellular responses to cigarette smoke exposure which inhibits the protective immunomodulatory effects of vitamin D [126]. There is research suggesting a genetic link between vitamin D and COPD pathogenesis. In an observational study single nucleotide polymorphisms in the vitamin D binding protein (VDBP) predicted vitamin D levels in COPD patients and were found to be a risk factor for COPD [123]. The VDBP is also involved in macrophage activation as high levels of airway VDBP are related to increased macrophage activation, also high levels of serum VDBP were found to be related to lower lung function [127]. COPD progression may also be affected by vitamin D status through absence of the vitamin D receptor and parenchyma degradation [128]. COPD exacerbations are generally caused by viral or bacterial lung infections, and though vitamin D has a positive role in reducing infection, there is no evidence to support that vitamin D is associated with ameliorating exacerbations in COPD patients [129]. The extra-skeletal effects of vitamin D are well documented in both asthma and COPD, and deficiency is associated with negative respiratory and immune outcomes. At this stage however, more evidence from supplementation interventions is needed before widespread adoption of supplementation can be recommended.

### 3.6. Minerals

Some minerals have also been found to be protective in respiratory conditions. In children, increased intake of magnesium, calcium and potassium is inversely related to asthma prevalence [7]. While several observational and experimental trials have been performed with conflicting results [130], a randomised controlled trial concluded that a low sodium diet had no therapeutic benefit for bronchial reactivity in adults with asthma [131]. Dietary magnesium may have beneficial bronchodilator effects in asthma [132]. Low dietary magnesium intake has been associated with negative effects on bronchial smooth muscle in severe asthma [133] and with lower lung function in children [134]. However further evidence of positive therapeutic effects are required before its importance in asthma and recommendations can be determined [135]. Dietary intake of selenium has been shown to be lower in asthmatics compared to non-asthmatics [136] and maternal plasma selenium levels were reported to be inversely associated with risk of asthma in children [137]. However case control studies in children have not found a relationship with selenium levels or intake with asthma related outcomes [18,138]. Furthermore, results from a large well designed RCT in adults with asthma showed no positive benefit of selenium supplementation [139]. Investigation of minerals in cord blood imply the importance of adequate intake during pregnancy, as levels of cord blood selenium were negatively associated with persistent wheeze, and levels of iron were negatively associated with later onset wheeze in children [140]. Studies on dietary intake of minerals and associations with COPD are sparse. A small study in Sweden found that in older subjects with severe COPD, intakes of folic acid and selenium were below recommended levels, and although intake of calcium was adequate, serum calcium levels were low, likely related to their vitamin D status as intake was lower than recommended [141]. Mineral intake may be important in respiratory diseases, yet evidence for supplementation is weak. It is likely that adequate intake of these nutrients in a whole diet approach is sufficient.

## 4. Obesity, Adipokines and Respiratory Disease

Overnutrition and resulting obesity are clearly linked with asthma, though the mechanisms involved are still under investigation. The review by Periyalil *et al.* [142] describes how immunometabolism-adipose tissue derived immunological changes causing metabolic effects [143] contributes to the link between asthma and obesity. In the obese state dietary intake of lipids leads to increased circulating free fatty acids [144], which activate immune responses, such as activation of TLR4, leading to increased inflammation, both systemically and in the airways [20]. Adipose tissue also secretes adipokines and asthmatic subjects have higher concentrations of circulating leptin than healthy controls [14] which are further increased in females, though leptin is associated with BMI in both males and females [145]. Leptin receptors are present in the bronchial and alveolar epithelial cells and leptin has been shown to induce activation of alveolar macrophages [146] and have indirect effects on neutrophils [147]. Also leptin promotes Th1 proliferation inducing increased activation of neutrophils by TNF-α [148]. *In vitro*, leptin also activates alveolar macrophages taken from obese asthmatics, which induces airway inflammation through production of pro-inflammatory cytokines [149]. However, a causal role for leptin in the obese asthma relationship is yet to be established. Adiponectin, an anti-inflammatory adipokine, has beneficial effects in animal models of asthma [150], however, positive associations in human studies have only been seen in women [151]. In obesity, macrophage and mast cell infiltration into adipose tissue is upregulated [142]. Neutrophils also appear to dominate airway inflammation in the obese asthma phenotype [152], particularly in females [153], which may explain why inhaled corticosteroids are less effective in achieving control in obese asthma [154]. While the mechanisms are yet to be understood, a recent review reports that obesity in pregnancy is associated with higher odds of asthma in children, with increased risk as maternal BMI increases [155].

COPD is characterised not only by pulmonary deficits but also by chronic systemic inflammation and co-morbidities which may develop in response to the metabolic dysregulation that occurs with excess adipose tissue [156]. A recent meta-analysis of leptin levels in COPD reported a correlation with body mass index (BMI) and fat mass percent in stable COPD though absolute levels were not different to healthy controls [157]. During exacerbation, leptin levels increased and were positively associated with circulating TNF-α [157]. Bianco *et al.* [158] describes the role of adiponectin and its effect on inflammation in COPD. Adiponectin has anti-inflammatory effects and is present in high concentrations in serum of healthy subjects [159]. Adiponectin exists in several isoforms, which have varied biological effects [160] and interact with two receptors present in the lungs (AdipoR1 and AdipoR2) that have opposing effects on inflammation [161]. Single nucleotide polymorphisms in the gene encoding adiponectin are associated with cardiovascular disease, obesity and the metabolic syndrome [162]. The role of adiponectin in COPD however is not well understood. In COPD, serum adiponectin is increased and directly relates to disease severity and lung function decline [163]. There is an alteration in the oligomerisation of adiponectin in COPD resulting in increased concentrations of the anti-inflammatory higher-molecular weight isoform [164], and the expression of adiponectin receptors in the lung is also altered in comparison to healthy subjects [165]. Animal models have shown anti-inflammatory effects of adiponectin in the lung through the increased expression of TNF-α in alveolar macrophages in adiponectin deficient mice [166]. Further mechanistic studies have also shown the anti-inflammatory potential of adiponectin by reducing the effects of TNF-α, IL-1β and NF-κB and increasing expression of IL-10 through interaction with AdipoR1 [161]. However under certain conditions in cell lines and animal models adiponectin has been shown to have pro-inflammatory effects [167,168]. As both detrimental and protective effects have been seen, the complex modulation of adiponectin isoforms and receptors in COPD requires further exploration. Obesity, the resulting systemic inflammation and alterations in adipokines have significant negative effects in both asthma and COPD. While work examining the mechanisms of effect is extensive, evidence for interventions to improve the course of disease are limited to weight loss interventions in asthma at this stage.

## 5. Undernutrition and Respiratory Disease

Though underweight has not been well studied in asthma, an observational study in Japan reported that subjects with asthma who were underweight had poorer asthma control than their normal weight counterparts [169]. While there is widespread acknowledgement that malnutrition in pregnant women adversely effects of the lung development of the fetus [170], a recent review reported that the offspring of mothers who were underweight did not have an increased risk of asthma. Amongst the obstructive lung diseases, undernutrition is most commonly recognised as a feature of COPD. Itoh *et al.* [171] present a review on undernutrition in COPD and the evidence for nutritional therapy in management of the disease. Weight loss, low body weight and muscle wasting are common in COPD patients with advanced disease and are associated with reduced survival time and an increased risk of exacerbation [172]. The causes of undernutrition in COPD are multifactorial and include reduced energy intake due to decreased appetite, depression, lower physical activity and dyspnoea while eating [173]. In addition, resting energy expenditure is increased in COPD, likely due to higher energy demands from increased work of breathing [174]. Also, systemic inflammation which is a hallmark of COPD, may influence energy intake and expenditure [175]. Cigarette smoke may also have deleterious effects on body composition in addition to the systemic effects of COPD. Smoking causes muscle fibre atrophy and decreased muscle oxidative capacity shown in cohorts of non-COPD smokers [176,177] and in animal models of chronic smoke exposure [178,179]. The mechanisms underlying muscle wasting in COPD are complex and multifaceted [180]. Increased protein degradation occurs in the whole body, though it is enhanced in the diaphragm [181]. Protein synthesis pathways are altered, indeed insulin like growth factor-1 (IGF-1) which is essential for muscle synthesis is decreased in cachectic COPD patients [182] and is lower in COPD patients during acute exacerbation, compared to healthy controls [183]. Increased oxidative stress, due to increased mitochondrial ROS production, occurs both systemically and in muscle tissue in cachectic COPD patients and is negatively associated with fat free mass (FFM) and muscle strength in COPD patients [184]. Furthermore myostatin induces muscle atrophy by inhibiting proliferation of myoblasts and mRNA expression of myostain is increased in cachectic COPD patients and is related to muscle mass [185]. Systemic inflammatory mediators such as TNF-α and NF-κB are also implicated in COPD muscle atrophy [186,187]. Nutritional supplementation therapy in undernourished COPD patients has been shown to induce weight gain, increase fat free mass, increase grip strength and exercise tolerance as well as improve quality of life [188]. Further studies point out the importance of not only high energy content, but also macronutrient composition of the nutritional supplement and inclusion of low intensity respiratory rehabilitation exercise [189,190]. Other dietary nutrients have been investigated for the benefits in COPD. Creatinine, found in meat and fish, did not have additive effects to rehabilitation, while sulforaphane, found in broccoli and wasabi, and curcumin, the pigment in turmeric, may have beneficial antioxidant properties [191,192,193]. Branched chain amino acid supplementation in COPD is associated with positive results including increases in whole body protein synthesis, body weight, fat free mass and arterial blood oxygen levels [194,195]. Undernutrition is not a significant problem in asthma, though is a major debilitating feature of COPD. There is promising evidence that nutritional supplementation in COPD is important and can help to alleviate some of the adverse effects of the disease, particularly muscle wasting and weight loss.

## 6. Conclusions

Dietary intake appears to be important in both the development and management of respiratory diseases, shown through epidemiological and cross-sectional studies and supported by mechanistic studies in animal models. Although more evidence is needed from intervention studies in humans, there is a clear link for some nutrients and dietary patterns.

**Figure 1:**
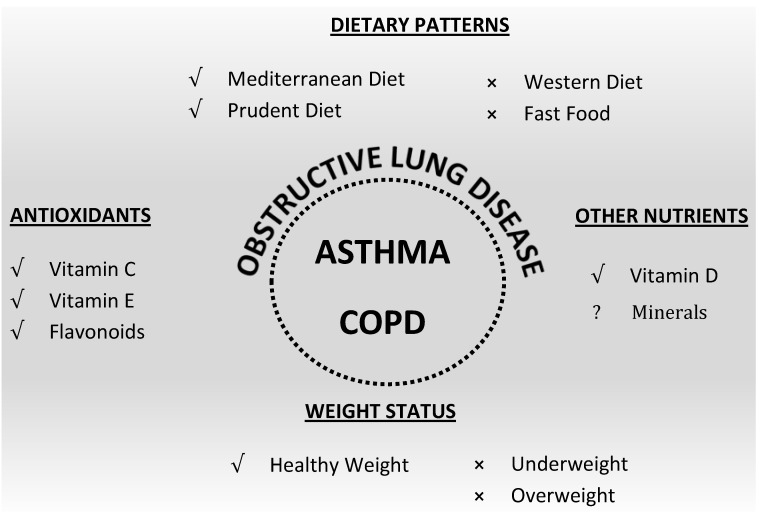
Relationship of Nutrition and Obstructive Lung Diseases: Dietary factors that have been linked to respiratory disease. √ evidence suggests positive effect, ⨯ evidence suggests negative effect, ? evidence is lacking.

The dietary patterns associated with benefits in respiratory diseases include high fruit and vegetable intake, Mediterranean style diet, fish and omega-3 intake, while fast food intake and westernised dietary patterns have adverse associations. Figure 1 shows a diagrammatic representation of the relationships of nutrition and obstructive lung diseases.

Though antioxidants are associated with positive effects on inflammation, clinical outcomes and respiratory disease prevention, intervention studies of individual antioxidants do not indicate widespread adoption of supplementation [196]. Differences in results from individual studies including whole foods such as fruit and vegetables and fish could be influenced by the nutritional profile owing to the region it was grown or produced. In considering studies using single nutrients it is also important to acknowledge that nutrients in the diet are consumed as whole foods that contain other micronutrients, fibre and compounds with both known and unknown anti and pro-inflammatory potential. Furthermore investigations of single nutrients should ideally control for other antioxidants and dietary sources of pro-inflammatory nutrients. While this limitation is common, it is a significant challenge to control for dietary intake of other nutrients in clinical trials. A whole foods approach to nutrient supplementation—for example, increasing intake of fruit and vegetables, has the benefit of increasing intake of multiple nutrients, including vitamin C, vitamin E, carotenoids and flavonoids and shows more promise in respiratory diseases in terms of reducing risk of COPD [3] and incidence of asthma exacerbations [25].

The evidence for mechanisms of vitamin D in lung development and immune function are yet to be fully established. It appears that vitamin D is important in respiratory diseases and infections, however the temporal role of vitamin D deficiency in disease onset, pathogenesis and exacerbations and whether supplementation is indicated is yet to be clarified.

Overnutrition in respiratory disease is clearly associated with adverse effects, highlighted by detrimental effects induced by immunometabolism. Further understanding of the relationship between mediators of immunometabolism and respiratory diseases and their mechanisms may provide therapeutic options. Undernutrition still poses risk in some respiratory conditions. Appropriate nutritional supplementation in advanced COPD is indicated, and several nutrients appear to be beneficial in COPD development and exacerbation.

The field of nutrition and respiratory disease continues to develop and expand, though further work is required in the form of randomised controlled dietary manipulation studies using whole foods to enable provision of evidence based recommendations for managing respiratory conditions.
